# Comparative Study of Essential Oils from Different Organs of *Syzygium cumini* (Pamposia) Based on GC/MS Chemical Profiling and In Vitro Antiaging Activity

**DOI:** 10.3390/molecules28237861

**Published:** 2023-11-30

**Authors:** Naglaa S. Ashmawy, Haidy A. Gad, Heba A. S. El-Nashar

**Affiliations:** 1 Department of Pharmaceutical Sciences, College of Pharmacy, Gulf Medical University, Ajman P.O. Box 4184, United Arab Emirates; 2 Department of Pharmacognosy, Faculty of Pharmacy, Ain Shams University, Cairo 11566, Egypt; haidygad@pharma.asu.edu

**Keywords:** antiaging, anti-collagenase, anti-elastase, essential oils, GC/MS, HCA, PCA, *Syzygium cumini*

## Abstract

*Syzygium cumini* L. is an evergreen tree belonging to family Myrtaceae, employed for different traditional uses like diabetes, inflammation, and fever. The current study aimed to compare the chemical compositions of the essential oils (EOs) isolated from different organs of *Syzygium cumini* (leaves (Scl), fruits (Scf), seeds (Scs), and bark (Scb)) using a GC/MS analysis. Also, a chemometric analysis was applied to explore the main similarities and differences among different organs using a Principal Component Analysis (PCA) and a hierarchal cluster analysis (HCA). Furthermore, in vitro antiaging activities were investigated via anti-collagenase, anti-elastase, and anti-hyaluronidase assays. The GC-MS analysis revealed 82 compounds representing 92.13%, 99.42%, 100%, and 92.97% in Scl, Scf, Scs, and Scb, respectively. The predominant components were α-pinene, β-pinene, (E)-β-caryophyllene, α-caryophyllene, caryophyllene oxide, and α-humulene epoxide II with variable percentages. All EOs were positioned on positive PC1, except for Scs, which was positioned on the negative side in a separate quadrant. The HCA dendrogram displayed the closeness of Scl and Scb, which was not clearly recognized in the PCA score plot. Moreover, the Scs oils were totally discriminated from other parts. The Scl and Scs oils showed superior anti-collagenase, anti-elastase, and anti-hyaluronidase activities. Thus, *S. cumini* oils should be considered for cosmetic preparations to retard skin aging manifestations.

## 1. Introduction

The aging process is a biochemical process caused by oxidative stress, which is attributed to endogenous free radicals, which leads to the progression of age-related manifestations [1]. Reactive oxygen species (ROS) or free radicals are reported to cause a change in the skin cell composition as well as damage to the cell membranes, leading to both DNA damage and cell death [2]. Furthermore, ROS play crucial roles in the aging process by destroying the skin’s major proteins like elastin and collagen via the activation of elastase and collagenase enzymes. Moreover, ROS causes the degradation of hyaluronic acid via the activation of hyaluronidases, which prevent proper skin hydration [3]. Antioxidants are the natural protection compounds found in the skin and scavenge the excessive free radicals in the body. When the load of ROS in the body is expressively higher than that of natural antioxidants, this leads to a condition called oxidative stress. Therefore, the use of external antioxidants from sources like diet or pharmaceuticals to neutralize ROS and counter the aging process is highly important [4]. Various studies have reported that natural products have many important biological roles such as antibacterial [5], antifungal [6], and anticancer activities [7], as well as their abilities to significantly contribute to the total antiaging and antioxidant activities in various plants [8]. This led to an increase in the interest to investigate antiaging in terms of the activities from different natural sources [9,10].

Plant essential oils are well known in traditional medicine as well as in aromatherapy for the management of numerous diseases [11,12,13,14]. Essential oils extracted from different plant sources were reported to possess antioxidant and antiaging properties [15,16]. Essential oils have also been reported as promising agents for the treatment of neurodegenerative diseases because they possess strong free radical scavenging activity, and hence, cause the inhibition of oxidative stress in the body [17,18].

*Syzygium cumini* L. Skeels (common name: Pamposia or Jamun) is an evergreen tree that belongs to the family Myrtaceae [19]. It is widely allocated in India and in Southeast Asian countries [20]. Traditionally, different parts of *S. cumini* have been employed for the treatment of various ailments. The leaves are utilized as remedies for indigestion, dysentery, stomach pain, and diabetes [21,22]. In addition. The leaves are applied as anti-inflammatory, antipyretic, and antiemetic agents [23,24,25]. However, the fruits have been used for gastric complaints as anti-flatulent and stomachic agents and for the treatment of dysentery [20]. Moreover, the fruits have been employed for the treatment of cough, inflammation, and diabetes [22,26]. Regarding *S. cumini* seeds, the powdered seeds have been described for dysentery and diabetes [20,27], and they have also been applied externally to cure sores and ulcers [28]. The decoction of the bark has been utilized to cure diabetes [29], dysentery, headache, and to improve appetite [26], in addition to the treatment of recurrent miscarriages [28].

Various studies have reported the effectiveness of the essential oil of *S. cumini* leaves as an antifungal [19,30,31], antibacterial, and antioxidant agent [31,32,33,34,35]. Moreover, the essential oil was reported to have molluscicidal, lishmanicidal [23,36,37], anti-inflammatory [38,39], larvicidal [40], and cytotoxic activities [37]. Few studies have been concerned with the biological effect of the essential oils of fruits, barks, and seeds, where most of the studies were conducted on the various extracts. Moresco et al. proved that the aqueous extract of *S. cumini* seeds have anti-inflammatory and antioxidant activities [41]. However, the alcoholic extract exhibited antioxidant and antipyretic activities [20]. Fruit extracts have been reported to exhibit anticancer, antioxidant [20,42,43], and hypoglycemic activities [44]. The aqueous, ethanol, and n-hexane extracts from the leaves, fruit, and bark of *Syzygium cumini* (L.) displayed antifungal activities [30]. The fruit pulp of *Syzygium cumini* (L.) Skeels has been reported for its protective effects against anxiety and dementia in aged mice [45], and this may be attributed to the plant’s antioxidant properties [46].

In light of prior research demonstrating the antioxidant properties of *Syzygium cumini* essential oils, this study not only aims to investigate the potential antiaging effects of essential oils obtained from *Syzygium cumini* leaves but also to comprehensively analyze the chemical composition and investigate the antiaging properties of oils extracted from other plant organs, including fruits, bark, and seeds. This study represents the first comparative metabolic analysis of essential oil compositions from various *Syzygium cumini* plant organs, utilizing GC/MS profiling coupled with chemometric analysis, specifically Principal Component Analysis (PCA) and Hierarchical Cluster Analysis (HCA), to identify the key similarities and differences among these plant organs. Furthermore, this study seeks to assess the antiaging potentials of these essential oils through anti-collagenase, anti-elastase, and anti-hyaluronidase inhibition assays.

## 2. Results and Discussion

### 2.1. GC/MS Analysis of the Essential Oils

A GC-MS analysis of the essential oils (EOs) isolated from different organs of *Syzygium cumini* could detect 82 compounds representing 92.13%, 99.42%, 100%, and 92.97% of *Syzygium cumini* leaves (Scl), fruits (Scf), seeds (Scs), and bark (Scb), respectively, as illustrated in Table 1. α-pinene, β-pinene, (*E*)-β-caryophyllene, α-caryophyllene, caryophyllene oxide, and α-humulene epoxide II were the main identified components in all of the studied organs with variable percentages. Caryophyllene oxide (17.24%) and α-terpineol (12.31%) are the major identified components in the leaf EOs, with small amounts of α-humulene epoxide II (8.27%) and bornyl acetate (5.85%). Regarding the EOs of fruits, the main detected compounds are caryophyllene oxide (22.63%), (*E*)-β-caryophyllene (13.6%), α-pinene (13.3%), α-terpineol (12.48%), α-caryophyllene (10.05%), α-humulene epoxide II (7.62%), and β-pinene (6.58%). However, (*E*)-β-caryophyllene (60.79%) and α-caryophyllene (25.15%) are the key components comprising the seed oil with little amounts of α-pinene (5.47%) and β-pinene (5.20%). For the stem bark, its essential oil is characterized by the presence of caryophyllene oxide (26.92%), α-humulene epoxide II (10.44%), α-pinene (7.29%), and α-terpineol (6.79%).

Our results were slightly different from that reported in the literature; this may be attributed to various factors such as the geographical source, harvesting period, method of oil isolation, environment, and growth conditions that may affect the essential oil composition [11,47]. In Egypt, various authors reported the leaf essential oil composition of *S. cumini*, where Elansary et al. reported α-pinene (17.53%), α-terpineol (16.67%), and alloocimene (13.55%) as its major components [33]. However, in other studies, the abundant constituents of the oils were α-pinene (32.32%), β-pinene (12.44%), and (*E*)-caryophyllene (11.19%) [34]. In accordance, Badawy et al. also recognized α-pinene (17.26%) and β-pinene (11.28%) as the main constituents in the leaf oil in addition to α-terpineol (13.88%) [31]. It was noticed that α-pinene, β-pinene, and (*E*)-caryophyllene were present in higher percentages in the literature when compared to our results; however, α-terpineol was closely related to the results reported in the literature. Also, a recent study carried out by El-Nashar et al. reported α-pinene (21.09%), β-(*E*)-ocimene (11.80%), D-limonene (8.08%), β-pinene (7.33%), and α-terpineol (5.38%) as predominant components [11].

Several studies reported the leaf essential oil constituents of *S. cumini* growing in Brazil, where α-pinene (31.85%), (*Z*)-β-ocimene (28.98%), and (*E*)-β-ocimene (11.71%) were the major detected ones [23]. However, α-caryophyllene (25.24%), β-caryophyllene (16%), and α-terpineol (9.08%), together with α-pinene (21.20%), globulol (15.30%), eugenol (11.20%), and α-terpineol (8.88%), were the key components [37] in other reported studies [38]. On the contrary, the leaf essential oil composition of *S. cumini* growing in India exhibited significant variations, where α-humulene (12.30%), in addition to β-caryophyllene (6.34%) and α-terpineol (5.71%), were the major detected constituents [48]. In another study, the principal components were α-pinene (21.5%), α-terpinessential oil (9.5%), *δ*-cadinene (8.3%), and (*E*)-ocimene (6.8%), [49]. Saroj et al. reported that α-pinene (17.2%), β-pinene (8.6%), (*Z*)-β-ocimene (10.9%), (*E*)-β-ocimene (9.6%), and *δ*-cadinene (7.5%) were identified as major constituents [19]. However, *τ*-cadinol (21.44%) and *τ*-muurolol (12.01%), globulol (7.98%), caryophyllene (6.69%), *δ*-cadinene (6.56%), and α-pinene (6.32%) were also found [50]. 

Concerning the fruits, (*Z*)-ocimene (29.95%), (*E*)-ocimene (23.03%), β-myrcene (6.99%), and α-terpineol (6.46%) were the major identified constituents in the essential oil isolated from the pulp [51]. Similarly, the major compounds identified in unripe fruit pulp were (*E*)-β-ocimene (25.59%), β-ocimene (16.83%), caryophyllene (20.31%), and humulene (12.83%) [52]. However, in another study, α-cadinol (25.8%), α-pinene (12.4%), β-pinene (8.0%), and myrcene (8.4%) were the principal components isolated [49].

### 2.2. Chemometric Analysis of the Essential Oils

**Figure 1:**
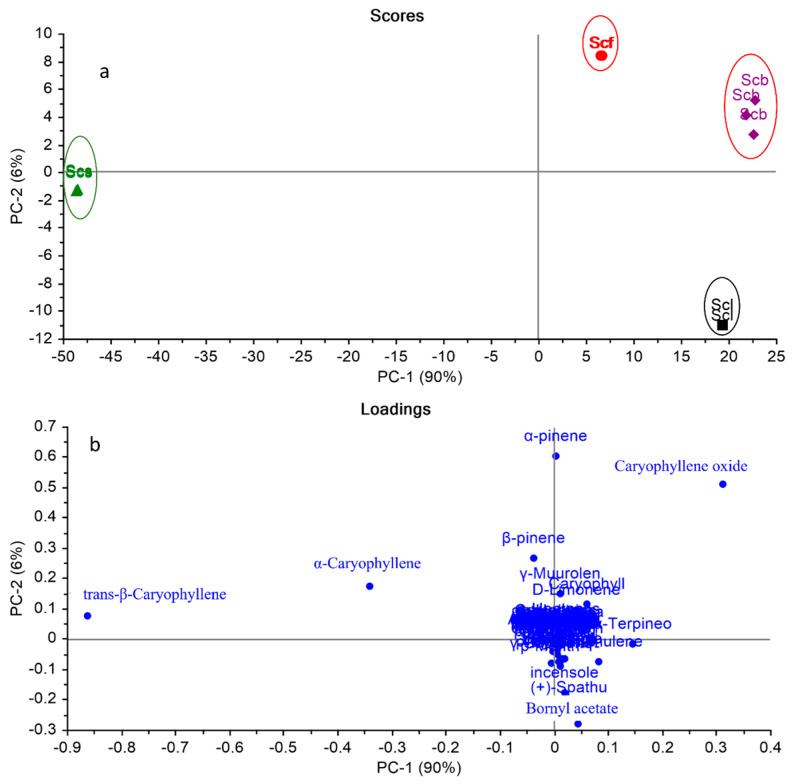
PCA score plot (**a**) and loading plot (**b**) based on GC-MS identification of the chemical compositions of the essential oils of various organs of *Syzygium cumini*. Abbreviations: (Scf) *S. cumini*, (Scl) *S. cumini* leaves, (Scf) *S. cumini* fruits, (Scs) *S. cumini* seeds, (Scb) *S. cumini* bark.

As observed in Table 1, the GC-MS data displayed both qualitative and quantitative variations among different organs that cannot be interpreted by the naked eye. The application of a multivariate analysis using a principal component analysis (PCA) and a hierarchal cluster analysis (HCA) enables the exploration of the similarities and differences between various samples based on the GC/MS data analysis [53,54]. A matrix of the total number of samples and their replicates (12 samples) multiplied by 82 variables (GC/MS peak area%) was constructed in MS Excel^®^ (Microsoft 365), and then exposed to multivariate analyses (PCA and HCA). Figure 1a,b display the PCA score and loading plots based on the GC-MS analysis of the chemical compositions of the essential oils of various organs of *S. cumini*, respectively.

The PCA score plot (Figure 1a) described about 96% of the data variance by the first two PCs, where PC1 explained 90% and PC2 explained 6%. Various *S. cumini* organs were grouped into four main clusters on three different quadrants. All *S. cumini* organs were positioned on positive PC1, except for the seeds that were positioned on the negative side in a separate quadrant. It was noticed that the *S. cumini* leaves (Scl) were placed on the lower right quadrant, completely discriminated from the other organs. Although the fruits (Scf) and the barks (Scb) were placed on the same quadrant (positive PC1 and PC2), they were clearly separated from each other. The loading plot (Figure 1b) displayed the key discriminating markers responsible for the PCA score plot pattern. (*E*)-β-Caryophyllene was the main marker responsible for the separation of *S. cumini* seeds (Scs) in a separate quadrant. Bornyl acetate and (+)-Spathulenol were the major components accountable for the segregation of leaves (Scl) in a single quadrant. Although caryophyllene oxide presents in approximately the same percentage in both Scf and Scb, it was observed that it applies a high load for the segregation of the barks only. However, α-pinene could separate Scf from the bark, although they were in the same quadrant. From the loading plot, it was observed that the key markers responsible for the segregation of various organs are not those that are present in high percentages, which proves the significance of the whole metabolic profile in the discrimination between various organs, and not only the ones that are identified in higher concentrations.

**Figure 2:**
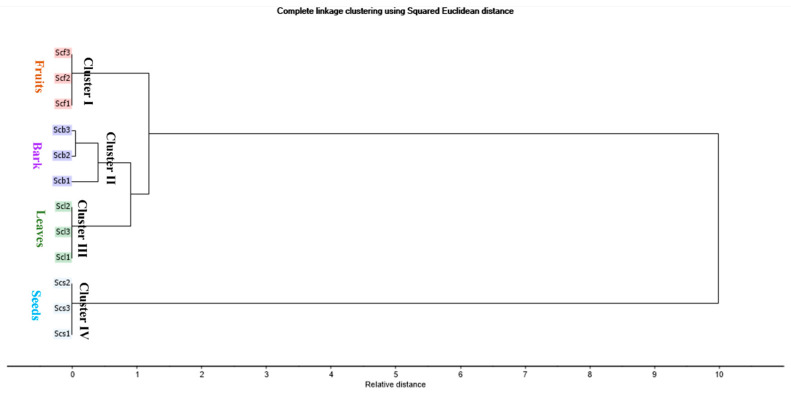
HCA dendrogram based on GC-MS identification of the chemical compositions of the essential oils of various organs of *Syzygium cumini* (leaves, fruits, bark stems, and seeds), as displayed in Table 1.

An HCA was applied to explore the closeness of various organs based on the relative measured distance. The dendrogram (Figure 2) revealed the segregation of various organs into four main clusters. Cluster I, II, III, and IV displayed Scf, Scb, Scl, and Scs, respectively. The HCA dendrogram disclosed the closeness of the barks and the leaves, which was not clearly recognized in the PCA score plot. Moreover, the HCA dendrogram confirmed that Scs was totally discriminated from the other parts.

### 2.3. Assessment of Anti-Collagenase, Anti-Elastase, and Anti-Hyaluronidase Activities

Collagen and elastin are vital protein components of the skin epidermis that play important roles in sustaining the elasticity of the skin [55]. Collagen makes up 70–80% of the skin’s dry weight and provides the dermis with structural and mechanical integrity. Although elastin is a minor component of the skin (2–4% of the extra cellular matrix), it has a significant role in providing the skin with its elasticity [56]. Elastin forms elastic fibers, which provide the stretch and recoil of the skin [57]. Elastin has a low rate of turnover, which causes it to be susceptible to loss over time [58]. Both collagen and elastic fibers show marked alterations with age in their three-dimensional arrangements. The synthesis of collagen reduces gradually, resulting in the decrease and disorganization in collagen in aged skin [59].

Skin aging is also caused by moisture loss in the skin. Hyaluronic acid is the key molecule involved in skin moisture with a unique ability to retain and bind water molecules [60]. The levels of elastin, collagen, as well as hyaluronic acid decrease gradually in the aging process, hence contributing to the loss of skin integrity and elasticity [61].

Many factors such as aging, the excessive exposure to UV light, as well as oxidative stress result in the activation of hydrolyzing enzymes like collagenase, elastase, and hyaluronidase, which will lead to the development of wrinkles and irregularities in the skin tone [62]. Therefore, the inhibition of collagenase, elastase, and hyaluronidase activities is one of the effective approaches to protect the skin from aging manifestations [63].

**Figure 3:**
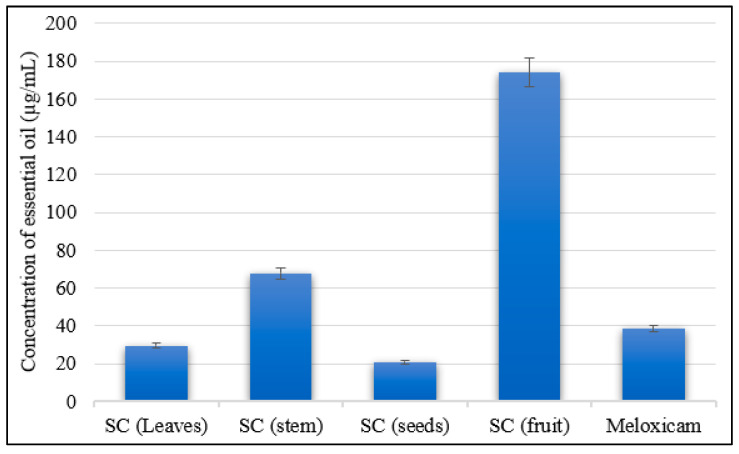
Collagenase inhibitory activity of the essential oils of various organs of *Syzygium cumini* (leaves, fruits, bark stems, and seeds).

As clarified in Figure 3, *S. cumini* seeds’ and leaves’ essential oils showed significant and remarkable anti-collagenase activities with IC_50_ values of 20.80 µg/mL and 29.39 n µg/mL, respectively, showing superior inhibitory effects compared to Meloxicam (IC_50_ = 38.48 µg/mL) as a standard drug. On the other hand, *S. cumini* bark essential oil showed moderate anti-collagenase activity with an IC_50_ value of 67.96 µg/mL, and *S. cumini* fruit essential oil exhibited weak anti-collagenase activity among all essential oil samples, with an IC_50_ value of 173.90 µg/mL, compared to the reference standard.

**Figure 4:**
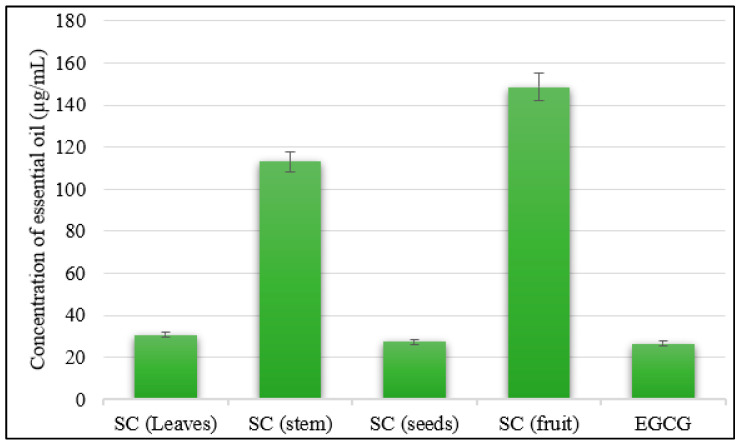
Elastase inhibitory activities of the essential oils of various organs of *Syzygium cumini* (leaves, fruits, bark stems, and seeds).

Similarly, the anti-elastase effects of *S. cumini* seeds’ and leaves’ essential oils showed significant results, with IC_50_ values of 27.44 µg/mL and 30.82 µg/mL, respectively, compared to epigallocatechin gallate (EGCG) (IC_50_ = 26.43 µg/mL) as the standard (Figure 4). On the other hand, the *S. cumini* stem bark and fruit essential oils showed lower inhibitions to the elastase effect, with IC_50_ values of 113.00 µg/mL and 148.50 µg/mL, respectively, compared to the reference standard.

**Figure 5:**
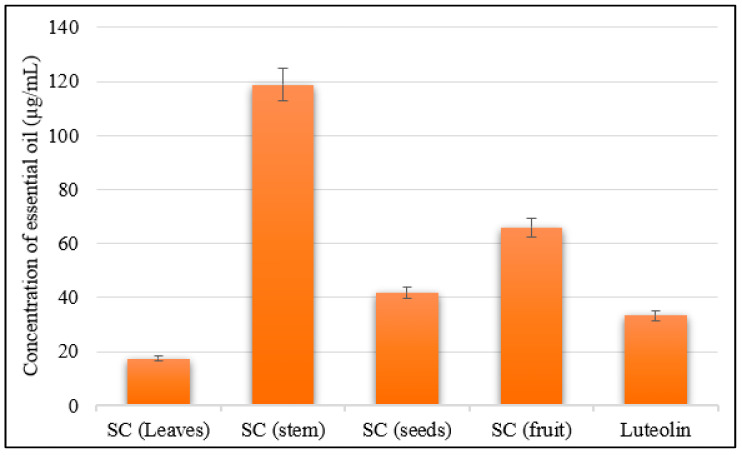
Hyaluronidase inhibitory activities of the essential oils of various organs of *Syzygium cumini* (leaves, fruits, bark stems, and seeds).

Moreover, among the four *S. cumini* essential oil samples, the leaf essential oil showed the highest inhibitory activity against the hyaluronidase enzyme, with an IC_50_ value of 17.35 µg/mL, showing greater inhibition compared to Luteolin (IC_50_ = 33.25 µg/mL) as a reference standard control. The seed essential oil exhibited a remarkable anti- hyaluronidase effect with an IC_50_ value of 41.74 µg/mL. The fruit oil showed a moderate anti-hyaluronidase effect with an IC_50_ value of 65.91 µg/mL, while the stem bark essential oil exhibited the least inhibitory effect (IC_50_ = 118.70 µg/mL) compared with the reference control (Figure 5).

In line with these results, a remarkable anti-collagenase activity of the essential oil isolated from another species of *Syzygium coriaceum* was previously reported with an IC_50_ value of 0.84 mg/mL [64]. Further prior studies have reported the antioxidant effects of *S. cumini* leaves’ essential oils (0.47 mg/100 mg) with a ferric reducing effect [34] and 1,1-diphenyl-2-picrylhydrazy (DPPH) radical scavenging activity, with a total antioxidant activity of 11.13% [33]. Further, a previous study reported the antioxidant activities of *S. cumini* fruits due to their richness in anthocyanins and carotenoids [65]. Moreover, *S. cumini* seeds were also reported for their antioxidant effects [66]. In addition to that, the antiaging activities of the essential oils derived from the leaves of the plant may be attributed to the high abundance of caryophyllene oxide (17.24%) in the oil; caryophyllene oxide was reported previously for its antiaging and antioxidant effects, where it showed strong cholinesterase inhibitory activity [67], and this was further evidenced by its significant binding interactions as well as docking scores with the acetylcholinesterase enzyme [68]. Moreover, the essential oil derived from the seeds of the plant showed significant anti-elastase as well as anti-collagenase activities, and this also may be attributed to the richness of the oil with β-caryophyllene (60.79%), which was reported for its antioxidant effects [69,70].

## 3. Materials and Methods

### 3.1. Plant Material

Different plant organs of *Syzygium cumini* (leaves, fruits, seeds, and bark stems) were collected in May 2022 from a private garden in the Masken Abo-Zabal region (N 30°17′43.386″, E 31°22′27.9804″), Qualiobya, Egypt. The plant was botanically identified by Therease Labib, the taxonomy specialist at the herbarium of the El-Orman Botanical Garden, Giza, Egypt. Voucher specimens were deposited with a code of PHG-P-SC-348 at the herbarium of the Department of Pharmacognosy, Faculty of Pharmacy, Ain Shams University, Cairo, Egypt. The symbols of Scl, Scf, Scs, and Scb are given to *Syzygium cumini* leaves, fruits, seeds, and bark stems, respectively.

### 3.2. Isolation of the Essential Oils

About 1 kg of different plant organs of *Syzygium cumini* (leaves, fruits, bark stems, and seeds) were subjected to hydrodistillation for 4 h using a Clevenger-type apparatus. The oils were collected, desiccated, and then stored at −4 °C in sealed vials, and protected from light for further analysis.

### 3.3. GC/MS Analysis of Essential Oils

The essential oil compositions of the different studied organs were analyzed via GC chromatograms and mass spectra using a Shimadzu GC/MS-QP 2010 (Kyoto, Japan) coupled to a mass spectrometer (SSQ 7000 quadrupole: Thermo-Finnigan, Bremen, Germany). A capillary column was applied (Rtx-5MS, 30 m length, 0.25 mm internal diameter, and 0.25 µm film thickness; Restek Co., Bellefonte, PA, USA. An initial temperature was adjusted at 45 °C for 2 min with gradual elevation to 300 °C at 5 °C/min for 5 min. The temperatures of the injector and detector were kept at 250 °C and 280 °C, respectively. Essential oil samples were diluted in *n*-hexane prior to analysis (1% *v*/*v*). Sample injection was applied automatically (1 µL, split ratio of 1:15). Helium carrier gas was utilized (flow rate of 1.41 mL/min). The mass spectrometer was carried out under the following conditions: ionization voltage of 70 eV, ion source temperature of 200 °C, and the scan range was adjusted from 35 to 500 *m*/*z*. Essential oil compositions were elucidated from their mass spectra and retention indices (RIs) relative to a mixture of a homologous series of standard *n*-alkanes (C_8_–C_28_) injected under the same conditions. The series of standard saturated *n*-alkanes (C_8_–C_28_) were obtained from Sigma-Aldrich Inc. (St. Louis, MO, USA), with a concentration of 1000 µg/mL for each component in hexane, and stored at 2–8 °C.

### 3.4. Identification of the Oil Components

The components of the essential oils were characterized via a comparison of their GC/MS spectra, fragmentation patterns, and retention indices with those reported in the literature [11,12]. The retention indices were calculated relative to a homologous series of *n*-alkanes (C_8_–C_28_) injected under the same conditions. These data were evaluated with NIST-11, Wiley Registry of Mass Spectral Database, and the data described in the literature [71,72]. 

### 3.5. Chemometric Analysis

The data obtained from GC-MS were subjected to chemometric analysis, where a matrix of the total number of samples (4 organs) and their replicates (4 × 3 = 12 samples) multiplied by 82 variables (GC/MS peak area % of the identified compounds) was constructed in MS Excel^®^, and then exposed to multivariate analyses (PCA and HCA). PCA was first employed as a primary step to explore the similarities and differences among different studied organs and to distinguish the markers responsible for this pattern. A PCA model was performed using cross validation method. Hierarchal cluster analysis (HCA) was then applied to allow for the clustering of different organs. The clustering pattern was created using the complete linkage method, applying squared Euclidean distance. PCA and HCA were accomplished utilizing Unscrambler^®^ X 10.4 software (Computer Aided Modeling, AS, Norway) [73,74].

### 3.6. Assessment of In Vitro Antiaging Activities

#### 3.6.1. Anti-Collagenase Activity

The inhibitory ability of *Syzygium cumini* essential oils against collagenase activity was tested using a fluorometric collagenase inhibitor screening kit (BioVision, Waltham City, MA, USA, catalog no. # K833-100) according to the method described previously [75]. Meloxicam was used as the reference control. The control and the tested oil samples were prepared in concentrations of 1, 10, 100, and 1000 μg/mL for the anti-collagenase analysis. A flat-bottom 96-well plate was used to prepare oil samples. The collagenase substrate was dissolved initially in Collagenase Assay Buffer (CAB). Then, preparation of the samples for analysis was performed by mixing them with collagenase as well as CAB. Inhibitor control samples were prepared by mixing the inhibitor Meloxicam (80 mM) with CAB buffer and the diluted collagenase enzyme. Preparation of the enzyme control was performed by mixing both diluted collagenase and CAB together. The background control used was the CAB buffer. All samples were then incubated at 25 °C for 15 min. During this time, mixing of the collagenase with CAB was performed to prepare the reaction mixture. In the next step, the prepared samples were mixed thoroughly with the reaction mixture. The emitted fluorescence was measured using a microplate reader (FilterMax F5, Thermo Fisher, Waltham, MA, USA) at 490 nm excitation wavelength and 520 nm emission wavelength. The fluorescence was measured in kinetic mode for 60 min at 37 °C. All samples were prepared in this method in duplicates, and the collagenase inhibitory activities of the tested samples were calculated by applying the following equation: % relative inhibition = [(enzyme control−sample)/enzyme control] * 100.

#### 3.6.2. Anti-Elastase Activity

*Syzygium cumini* oil samples were investigated for their anti-elastase activities using EnzChek^®^ Elastase Assay Kit (E-12056, Thermo Fisher, Waltham, MA, USA) according to the method reported previously [76]. The oil samples were prepared in clear-bottom 96-well plates for fluorometric assay. Elastase substrate, elastase enzyme solutions, in addition to the inhibitor control were prepared as per the described method. Diluted elastase solution was firstly added to the wells. Tested oil samples, enzyme control, as well as inhibitor control (Meloxicam) were added to the subsequent wells. All samples were mixed using a shaker and then incubated at 37 °C for 5 min. The fluorometric reaction mix was prepared by mixing the assay buffer with the substrate, which was added and then mixed with each sample. The fluorescence was measured using a microplate reader (FilterMax F5, Thermo Fisher) at 400 nm excitation wavelength and 505 nm emission wavelength. Measurement of the emitted fluorescence was conducted in kinetic mode for 30 min at 37 °C with protection from light. All measured samples were prepared in duplicates, and the anti-elastase inhibitory of the samples was calculated by applying the following equation: % relative inhibition = [ΔRFU (test inhibitor)/ΔRFU (EC)] * 100.

#### 3.6.3. Anti-Hyaluronidase Activity

The anti-hyaluronidase activity was evaluated spectrophotometrically by assessing the N-acetylglucosamine produced from sodium hyaluronate [77]. A total of 50 µL of bovine hyaluronidase (7900 units mL^−1^, Sigma, Burlington, MA, USA) prepared in 0.1 M acetate buffer was mixed with 100 µL of a designated concentration of the oil sample dissolved in 5% DMSO. In the following step, the prepared reaction mix was then incubated in a water bath for 20 min at 37 °C. The control group was prepared via treatment with 100 µL of 5% DMSO instead of the sample. Luteolin was used as a reference control. The reaction mixture’s optical density was measured spectrophotometrically at 585 nm.

### 3.7. Statistical Analysis

All analyses were carried out in triplicate. Values are conveyed as means ± SD. Statistical significance was determined using one-way ANOVA followed by Tukey’s post hoc test (significance level at *p* < 0.05).

## 4. Conclusions

As a conclusion, we described a detailed comparative study on the chemical compositions of the essential oils isolated from different organs of *S. cumini* grown in Egypt. All tested oils were found to be plentiful in α-pinene, β-pinene, (E)-β-caryophyllene, α-caryophyllene, caryophyllene oxide, and α-humulene epoxide II. Further, we explored the main similarities and differences among different organs using multivariate chemometric analyses (PCA and HCA). On the biological side, the leaf and seed essential oils exhibited superior inhibitory activities against the enzymes involved in the aging process such as collagenase, elastase, and hyaluronidase, making these essential oils and their major constituents good candidates in adjuvant therapy for anti-skin-aging cosmetic preparations. Moreover, in vivo studies on anti-wrinkle activity are recommended along with toxicity, pharmacokinetics, and pharmacodynamics studies to assemble a molecular mechanistic profile for the isolated essential oils in the management of skin aging, encouraging the utilization of *S. cumini* essential oils as herbal pharmaceutical products.

## Figures and Tables

**Table 1:** Chemical compositions of the essential oils based on GC/MS analysis (n = 3).

No.	KI		Compound	Relative Abundance%			
	Cal.	Rep.		Scl	Scf	Scs	Scb
1.	932	939	α-pinene	0.69	13.13	5.47	7.92
2.	946	946	Camphene	-	0.55	-	0.21
3.	972	971	α-thujene	0.86	-	-	-
4.	974	974	β-pinene	0.45	6.58	5.20	3.34
5.	991	991	β-Myrecene	-	0.42	-	0.06
6.	1024	1028	o-Cymene	0.48	-	-	0.36
7.	1028	1029	D-Limonene	1.69	2.67	-	4.08
8.	1059	1060	γ-terpinene	0.21	-	-	-
9.	1071	1071	Linalool oxide	-	-	-	0.05
10.	1097	1097	α-Pinene epoxide	-	-	-	0.34
11.	1100	1100	Linalool	0.48	-	-	0.7
12.	1106	1105	trans-para-Mentha-2,8-dien-1-ol	0.44	-	-	0.46
13.	1113	1111	Fenchol	1.12	0.62	-	0.68
14.	1122	1122	cis-2-p-Menthen-1-ol	0.28	-	-	0.20
15.	1126	1125	α-Campholenal	0.13	-	-	0.17
16.	1134	1134	Limonene 1,2-epoxide	-	-	-	0.12
17.	1139	1139	(-)-trans-Pinocarveol	0.67	0.5	-	0.78
18.	1145	1143	cis-Verbenol	0.75	-	-	0.27
19.	1148	1148	Camphene hydrate	0.77	-	-	0.41
20.	1163	1164	Pinocarvone	0.12	0.43	-	0.31
21.	1166	1166	Endo-Borneol	0.85	0.57	-	0.59
22.	1178	1182	Terpinen-4-ol	1.60	-	-	0.45
23.	1187	1188	p-Cymen-8-ol	1.48	-	-	0.22
24.	1193	1192	α-Terpineol	12.31	12.48	-	6.79
25.	1198	1198	Myrtenal	0.73	0.75	-	0.70
26.	1210	1210	Verbenone	0.40	-	-	0.29
27.	1221	1220	α-Fenchyl acetate	1.45	-	-	0.58
28.	1224	1227	2-Hydroxy-1,8-cineole	0.32	-	-	0.06
29.	1243	1243	p-Cumic aldehyde	0.22	-	-	-
30.	1247	1246	Carvone	0.11	-	-	0.25
31.	1277	1274	p-Menth-1-en-7-al	1.75	-	-	0.12
32.	1287	1287	Bornyl acetate	5.85	-	-	1.5
33.	1294	1293	p-Cymen-7-ol	0.23	-	-	-
34.	1301	1297	trans-Pinocarvyl acetate	0.42	-	-	0.52
35.	1306	1305	Carvacrol	0.61	-	-	0.39
36.	1351	1351	α-Terpinyl acetate	0.14	-	-	-
37.	1379	1379	α-Copaene	0.17	-	-	0.1
38.	1385	1385	Geranyl acetate	-	-	-	0.22
39.	1423	1422	trans-β-Caryophyllene	1.35	13.6	60.79	0.34
40.	1431	1432	α-Ionone	0.19	-	-	0.16
41.	1443	1447	Aromandendrene	0.1	-	-	0.15
42.	1458	1459	α-Caryophyllene	1.33	10.05	25.15	0.38
43.	1466	1466	Alloaromadendrene	0.31	-	-	-
44.	1474	1479	Aristolochene	0.53	-	-	0.20
45.	1480	1480	γ-Muurolene	0.33	4.04	-	0.15
46.	1492	1492	β-Selinene	0.76	-	0.51	0.15
47.	1504	1504	α-Muurolene	0.16	-	-	0.71
48.	1528	1528	γ-Cadinene	1.56	-	1.14	0.47
49.	1559	1552	Isopatchoulane	-	-	-	0.76
50.	1567	1567	trans-(E)-Nerolidol	1.03	-	-	0.23
51.	1577	1581	Palustrol	1.93	0.60	-	0.58
52.	1586	1586	(+)-Spathulenol	4.49	-	-	0.32
53.	1592	1592	Caryophyllene oxide	17.24	22.63	1.27	26.92
54.	1599	1598	Viridiflorol	0.32	-	-	0.41
55.	1609	1608	Epiglobulol	2.39	1.26	-	0.58
56.	1618	1613	α-Humulene epoxide II	8.27	7.62	0.46	10.44
57.	1640	1641	Caryophylla-4(12),8(13)-dien-5α-ol	1.59	0.88	-	1.55
58.	1644	1644	Caryophylla-4(12),8(13)-dien-5β-ol	0.62	-	-	0.87
59.	1648	1648	τ-Cadinol	0.38	-	-	0.55
60.	1653	1652	δ-Cadinol	0.1	-	-	0.14
61.	1659	1655	trans-Guai-11-en-10-ol	1.49	-	-	1.41
62.	1678	1679	α-Bisabolene oxide	0.76	-	-	2.1
63.	1731	1733	(Z)-α-Bisabolene epoxide	0.54	-	-	1.05
64.	1744	1746	Bisabolone	0.22	-	-	0.47
65.	1785	1789	β-bisabolen-15-ol	0.43	-	-	0.63
66.	1839	1838	Hexahydro farnesyl acetone	-	-	-	0.31
67.	1971	1961	Cembrene A	0.34	-	-	-
68.	2026	2033	Kaur-16-ene	0.49	-	-	-
69.	2084	2085	1-Octadecanol	-	-	-	0.07
70.	2097	2100	*n*-Heneicosane	-	-	-	0.1
71.	2114	2114	Phytol	0.27	-	-	-
72.	2128	2120	Phenethyl anthranilate	0.28	-	-	0.34
73.	2155	2161	Cembrenol	0.32	-	-	-
74.	2169	2158	incensole	3.41	-	-	-
75.	2197	2200	n-Docosane	-	-	-	0.13
76.	2297	2300	*n*-Tricosane	0.13	-	-	0.43
77.	2303	2302	Methyl cis-11-eicosenoate	0.12	-	-	-
78.	2397	2400	*n*-Tetracosane	-	-	-	0.32
79.	2496	2500	*n*-Pentacosane	0.2	-	-	0.87
80.	2696	2700	*n*-Heptacosane	-	-	-	1.53
81.	2895	2900	*n*-Nonacosane	0.21	-	-	1.77
82.	3095	3100	*n*-Hentriacontane	-	-	-	1.38
Total identified compounds%				92.13%	99.42%	100%	92.97%

Compounds were identified based on the compounds’ mass spectral data and retention indices compared with those of the NIST Mass Spectral Library (December 2011), the Wiley Registry of Mass Spectral Data, 8th edition, and many authentic standards. The content (%) was calculated in triplicate using the normalization method based on the GC-MS data. The presented data are shown as the average of three replicas. (-): Unidentified in the sample. Standard deviation did not exceed 3% for any of the values. KI: Kovats index calculated on Rtx-5MS column. (Scl) *S. cumini* leaves, (Scf) *S. cumini* fruits, (Scs) *S. cumini* seeds, (Scb) *S. cumini* bark.

## Data Availability

Data are available upon request from the corresponding authors: dr.naglaa@gmu.ac.ae (N.S.A.) and heba_pharma@pharma.asu.edu.eg (H.A.S.E.-N.).
